# Dietitians and Nutritionists: Stigma in the Context of Obesity. A Systematic Review

**DOI:** 10.1371/journal.pone.0140276

**Published:** 2015-10-14

**Authors:** Franziska U. C. E. Jung, Claudia Luck-Sikorski, Nina Wiemers, Steffi G. Riedel-Heller

**Affiliations:** 1 Institute of Social Medicine, Occupational Health and Public Health (ISAP), University of Leipzig, Leipzig, Germany; 2 Leipzig University Medical Center, IFB Adiposity Diseases, Leipzig, Germany; GDC, GERMANY

## Abstract

**Aim:**

Negative attitudes towards people with obesity are common even in health care settings. So far, the attitudes and causal beliefs of dietitians and nutritionists have not been investigated systematically. The aim of this article was to review the current state of quantitative research on weight-related stigma by dietitians and nutritionists.

**Method:**

A systematic literature review was conducted in 2014 using PubMed, PsycINFO, Web of Science and Cochrane Library.

**Results:**

Eight studies were found that differ in regard to study characteristics, instruments and the origin of the sample. Six out of eight studies reported weight stigma expressed by dietitians and nutritionists. Their believed causes of obesity indicated a defined preference for internal factors rather than genetics or biology.

**Discussion:**

Results of studies were not homogenous. The degree of negative attitudes by dietitians and nutritionists towards people with obesity appeared to be slightly less pronounced compared to the general public and other health care professionals. Stigma and its consequences should be included into educational programs to optimally prepare dietitians and nutritionists.

## Introduction

According to the World Health Organization (WHO), overweight and obesity have become a tremendous threat to the general population worldwide. Overweight and obesity are multifactorial conditions, which can be linked to a variety of genetic, hormonal or environmental causes. Many factors, such as socio-cultural (e.g. food environment, walkability), biophysical (e.g. genetics and neuroendocrinology), psychological (e.g. depression and stress) and medication-related factors can contribute to an increased energy intake and a lowered energy expenditure [1]. Besides having negative physiological consequences on well-being and health [2], this issue also transcends to the social level.

Weight stigmatization and exclusion processes have been found to increase rapidly during the last years [3]. Negative stereotypes such as laziness, not being motivated or a lack of self-discipline are often associated with people with overweight or obesity [4]. One reason for stigma of obesity can be found in the assumption that overweight can be personally controlled and therefore those affected by overweight are responsible themselves [5–7]. By neglecting biological, genetic as well as environmental causes of obesity, blame is increased on those affected, leading to negative attitudes towards individuals with overweight and obesity. Hence, the investigation of the causes of weight-related stigma is very important, in order to understand how prejudice related to obesity arise and how they can be overcome.

Weight stigmatization is very common among the general population [5]. Additionally, it has been shown that weight-related stigma is a serious issue, affecting the patients’ physical health, but also their mental health [8, 9]. Weight stigmatization has also been found to be a predictor of unhealthy eating [10] and a lack of physical activity [11, 12]. About 80% of participants (men and women) reported “eating” as a coping strategy in response to weight-related stigma and approximately three-quarters of them reported “refusing to diet” in order to cope with stigma [10]. A feedback loop model described by Tomiyama [13] aims to illustrate how stigma can lead to the undesirable effect of putting on weight. In this model, weight-related stigmatization can be seen as a stressor, which leads to increased cortisol levels and increased eating, hence resulting in weight gain, which in turn provokes more stigma and teasing.

Surprisingly, one of the biggest sources of weight-related stigma can be observed in health care areas [14, 15]. Previous studies indicated that health care professionals such as doctors, nurses and psychologists showed generic prejudice towards obesity, holding the view that the reason for extreme overweight was due to personal misconduct [16, 17]. From the patient’s perspective, physicians have been found to be the most frequent source of stigma for women and the second most frequent source of stigma for men [10]. This study also states that 37% of patients experienced weight bias by dietitians and nutritionists. Even if this is less compared to weight bias by doctors (69%), it confirms that weight stigma among this group exists and should not be undermined.

Weight stigmatization in health care can result in impaired outcomes for patients with obesity. Some studies have linked a high BMI to avoidance of health care prevention services or cancellation of appointments due to weight concerns (e.g. [18–22]). Especially women seemed to be prone to this kind of treatment avoidance due to concerns of being stigmatized due to their weight [21, 23]. Delaying necessary prevention checkups and treatment may contribute to the negative health outcomes we see in individuals with obesity [24].

There is additional empirical evidence, that weight-related bias can also negatively affect treatment seeking in terms of weight reduction [25, 26]. Patients who expect stigmatization from their health care provider may delay or even cancel attempts to seek help for weight reduction. Taken together, these negative consequences might explain why weight-related stigmatization makes it even harder for those affected to reduce weight and improve their health condition.

Apart from physicians, psychologists and nurses, another occupational category, which is intensively in contact with patients with overweight or obesity, has been rarely looked at in the past. Dietitians spend a lot of time with people with obesity or overweight and play a very important role in the management of obesity. Dietitians see themselves as the specialist contact person in the field of obesity management [27], which again expressively underlines the significance of this topic.

A previous review [4] reviewed stigmatization of individuals with obesity in great detail; however, the authors were only able to summarize a small number of studies addressing dietitians in particular, and were not able to include studies that assessed the dietitians’ belief of causes of obesity. Therefore, this review aims to complete and extend the current state of knowledge by (a) determining the magnitude of stigmatization of patients with obesity among dietitians and (b) summarizing causal beliefs of dietitians.

## Method

### Search Strategy

A systematic search of the literature on attitude of dietitians towards adiposity was conducted using four electronic databases: PubMed, PsycINFO, Web of Science and Cochrane Library. This review followed the Prisma Guidelines [28].

The following key words “obes* or adiposity or overweight or over-weight or fat; attitude or belief or prejudice or stigma or perception; as well as health care professionals or dietitian or dietitian or nutritionist” were used. Due to a very high number of results, the search was limited to title and abstract of the publications and only work published in English or German was included. The search was also limited to “human” studies and language was restricted to either “German” or “English”. No restriction regarding the year of publication or publication status was imposed. In order to overcome publication bias, all relevant studies that covered the topic under investigation were included as well as grey literature. This approach follows recommendation by the PRISMA Guidelines [28] and recommendations stated in the Cochrane Handbook for Systematic Reviews of Interventions [29]. Further details are given in S1 Table. The possibility of publication bias was additionally assessed by following the advice given by HLWIKI Canada [30] using the search engine *Google* in order to search for any grey literature such as dissertations or unpublished material that is related to the research question in accordance with the specific exclusion criteria described below. Neither Google web search (1100 results) nor Google Scholar (30 results) was useful to obtain unpublished material that was suitable for inclusion.

### Data extraction

In 2014, two reviewers conducted the search independently using a data extraction sheet as recommended in the literature [31]. Titles and abstract were assessed for eligibility and full papers were obtained. Out of 1,090 publications, 1,000 studies were excluded according to title and abstract. All abstracts with disagreement between the reviewers were re-visited again and agreement was found by discussion and consensus, screening articles in more detail in case there was uncertainty. In addition to the remaining 90 articles, two additional studies were chosen from the reference list of other articles. Overall, 92 studies were screened in full text using the following exclusion criteria: (i) other professions such as physicians, nurses or psychologists; (ii) studies that investigated stigma from the patient perspective; (iii) studies that were interested in more general opinions by dietitians, e.g. about treatment success and (iv) reviews or qualitative studies. In summary, 32 studies were excluded because their scientific focus did not fit into the exclusion criteria matrix, 34 studies included HCPs in general or did not explicitly differentiate between dietitians and other HCPs. Five studies were excluded because they were only interested in the patients’ perspective. In terms of methodological content, one study was excluded due to their method of analysis, seven studies were excluded because they were using qualitative methods and five studies were excluded because they were categorized as reviews. The different stages of this literature search are provided in S1 Fig.

**Fig 1:**
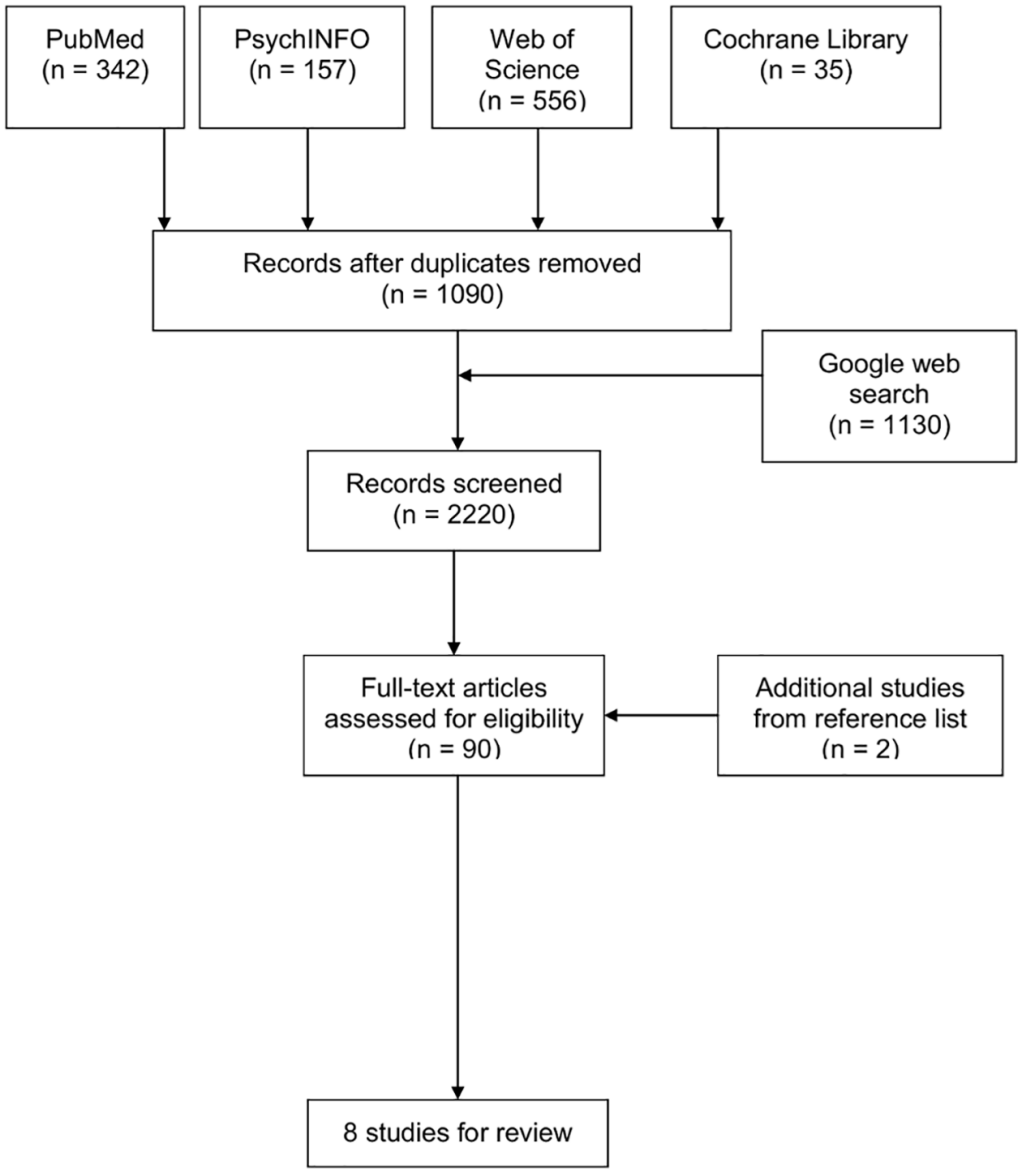
The different phases of the systematic review. ^1^HCP = Health Care Professionals.

After applying these criteria, eight studies were identified and be considered of importance in order to outline the current stage of research that has been done to investigate the topic up to now (Fig 1). Methodical data on sampling, design of the study, constructs under investigation as well as outcome criteria (measures of attitudes of dietitians and nutritionists) were extracted systematically by one reviewer and checked by a second reviewer independently. The remaining studies that were found to be eligible for detailed analysis were then tabulated according to the following characteristics: origin of the sample (Country); size of the sample (N); levels of qualification of participants under investigation (Sample); measuring scale (Instruments- explicit or implicit); as well as summary of results and connotation of attitudes (Table 1).

**Table 1:** Summary of methodological differences of all eight studies.

Author/ Country	N	Sample	Data Collection	Instrument	Results	Connotation of attitudes
Berryman, Duable, Manchester, Mittelstaedt (2006); USA[32]	76	38 female dietetic students (Ø age:21.2 years) and 38 female students from other departments (Ø age:21.4 years), studying at the University of Ohio	Experimental control group design; Explicit measures by use of questionnaires	Fat Phobia Scale	Explicit attitude: overall no significant difference between groups, dietetics students showed negative attitude towards obesity (Ø FPS = 3.66, ranging from 2.0 to 5.0), approx. 16.0% from both groups adopted strong negative attitude (Ø FPS≥4.4), 13.0% of dietetics majors showed neutral, slightly positive attitude (FPS<2.5)	Negative
Edelstein, Silva & Mancini (2009); USA[33]	128	Registered dietitians of the “American Dietetic Association” Gender: 99.0% female. Work experience: 5 years:61.0%; 2–5 years:30.0%; <1 year:9.0%	Implicit measures	Fat People-Thin People Implicit Association Test	Implicit attitude: 76.0% of dietitians showed a strong to moderate preference for thin people compared to obese people. Their own weight, age and origin did not have an impact on implicit attitude towards people with obesity.	Negative
Harvey, Summerbell, Kirk & Hill (2002); UK[34]	187	Randomly selected dietitians of the”British Dietetic Association”	Independent Measures Survey; Explicit measures by use of questionnaires	Questionnaire on Obesity and Overweight 1. Assumption about reasons for obesity (Harvey & Hill, 2001) 2. Attitude towards obesity (Harvey & Hill, 2001; Allison’s Attitude Towards Obese People, ATOP) 3. attribution of responsibility (Harvey & Hill, 2001)	1. Causes: generally no difference between Obesity and Overweight questionnaire, physical inactivity, caloric intake of unhealthy food to high, higher caloric intake due to mood changes, weight changes due to repetition of dieting, interpersonal factors. 2. Explicit attitude: overall neutral to positive; obese (BMI≥30.0) people were rated more negative in comparison to overweight (BMI 25.0–30.0) people; obese people were thought to be less successfully working, negatives attitude relate to an adopted negative self-esteem, low sexual attractiveness and poorer health. 3. Responsibility: overweight and (especially) obese people were seen as being responsible for their excess weight	Positive
Hellbardt, Riedel-Heller & Sikorski (2014); GER[35]	49	Randomly selected dietitians, participating at a congress on nutrition	Explicit measures by use of questionnaires	Vignette-based approach (two vignettes:42-years-old woman, either normal weight or over-weight) and the Fat Phobia Scale (14 pairs of adjectives)	Explicit attitude: statistical more negative evaluation of the vignette “over-weight”, especially for the following pairs “shapeless-shapely”, “secure-insecure” and “poor self-esteem–self-esteem”, FPS = 3.35 (overweight), compared to FPS = 2.61 (normal weight). Causes: most agreement towards internal causes of over-weight (e.g. lack of physical activity or overeating) compared to other reasons (genetic factors, condition-related reasons)	Negative
McArthur & Ross (1997); USA[36]	411	Registered dietitians of the “Presidents of State Dietetic Associations” (overall 439, 411 dietitians counsel overweight patients)	Explicit measures using questionnaire, contact via email	1.15 “attitude statements” about own weight 2.37 “attitude statements” about overweight (Men: BMI>26.4 and women: BMI>25.8) clients (rating scale from “strong agreement”, “neither nor” to “strong disagreement”)	Explicit attitude: Dietitians with (self-perceived) obesity showed negative attitude towards themselves (feel physical unattractive, blamed themselves for being obese). However, positive attitude related to own goal setting, preservation of weight, willpower>self-stigmatization. Ambivalent attitude towards obese clients. Attribution/ Reasons: Emotional problems; unrealistic goal setting, feelings of ambivalence in relation to obese people’s discipline to hang on a diet	Neutral
Oberrieder, Walker, Monroe & Adeyanju (1995); USA[37]	298	64 students studying Nutrition Science (Kansas State University); 234 registered dietitians (members of the Kansas Dietetic Association)	Explicit measures by use of questionnaires (send by post)	Bray Attitude Towards Obesity Scale, 47 items including a four-point Likert Scale, Range: positive attitude: 0.0 to 93.0; negative attitude: 94.0 to 188.0	Explicit attitude: Registered dietitians as well as students demonstrated negative attitude towards people with obesity (men: BMI≥27.3 and women: BMI≥27.8), participants with a self-reported healthful weight had a slightly more negative attitude compared to overweight participants Students: BATOS = 101.94 Dietitians: BATOS = 103.71	Negative
Puhl, Wharton & Heuer (2009); USA[38]	182	182 students studying nutrition science. Age: Ø23.1 years; degree program: since Ø1.7 years; gender: 92.0% female; weight (BMI): 80.0% normal weight (18.5–24.9), 5.0% underweight (<18.5), 14.0% overweight (25.0–29.9), 1.5% obese (30.0–39.0)	Randomized Experimental Study, Between-Subjects Design, questionnaire includes four different conditions/ patient profiles: 1. non-obese, female patient 2.non-obese, male patient 3.obese, female patient 4.obese, male patient	1. Fat Phobia Scale and 2. perception of the patient depending on test condition: How receptive is the patient in terms of dietary advice for treatment? Does he/she understand it? Compliances? Motivation? Patient’s ability to change or maintain weight? How much pleasure would it give me to work with this patient?	Explicit attitude: 1. Fat Phobia Scale: no differences between groups, all students showed moderate extent of fat phobia (ØFPS = 3.7), similar to the general population. 2. obese people were attributed less compliances than non-obese patients; quality of diet and medical condition of obese patients was rated poorer compared to people with normal weight	Negative
Swift, Hanlon, El-Redy, Puhl & Glazebrook (2013); UK[39]	1130	Students: “Master of Nutrition”, “Nursing”, B.Sc. in Nursing, B.Sc. in Medical Science, B.Sc. in Nutrition and Food Sciences, University of Nottingham	Explicit measures by the use of questionnaires	1. Fat Phobia Scale: individual adjectives not given. 2. Beliefs about Obese People: six-point scale, scores range from 0 to 48, the higher the score the greater the belief that obesity cannot be personally controlled.	Explicit attitude: 1.FPS = Ø3.8; 1.4% showed positive or neutral attitude; 10.5% showed more distinct signs of fat phobia. Signs of less marked fat phobia: a) higher BMI; b) B.Sc. in Nursing; c) a stronger belief that obesity cannot be personally controlled. 2.BAOP = Ø13.4	Negative

Note: Negative = demonstrates negative attitude towards adiposity; Positive = positive or neutral attitude towards adiposity; Neutral = ambivalent attitude or ambiguous findings; N = sample size; FPS = Fat Phobia Scale; IAT = Implicit Association Test; BATOS = Bray Attitude towards Obesity Scale; BAOP = Beliefs About Obese People

## Results

### Study characteristics

The methodological characteristics of the eight studies are summarized in Table 1.

Five out of eight studies were based on an American sample, whereas two studies came from Great Britain [34, 39] and only one study was based on a German sample [35]. Sample size varied between 49 [35] and 1,130 participants [39].

The studies also varied among the participants’ level of qualification. Four studies included practicing or registered dietitians who already gained work experiences, three studies surveyed participants studying dietetics or nutritional science and one study included both, students and practicing dietitians.

Women as well as men were equally considered in the majority of studies; however, more women were included in the samples. The study by Berryman et al. [32] should be named as an exception, investigating only female participants.

### Instruments

Seven studies overall examined dietitians’ attitude concerning obesity, using questionnaires as an explicit measurement. The study by Edelstein and colleagues [33] measured weight stigma on an implicit level using the Implicit Association Test (IAT).

Further differences between the studies could be found by looking at the type of explicit questionnaires and scales, measuring (over)weight-based attitude and prejudice. Seven studies used Adjective Check Lists, such as the Fat Phobia Scale (FPS) with five-point rating scale questions (four studies) or a semantic differential with characteristics and a seven-point rating scale [33]. The Fat phobia scale scores can range between 1 and 5 (1 = positive attitudes, 5 = negative attitudes). A score of 3.6 can be seen as moderately fat phobic and a score greater than 4.4 indicates high levels of fat phobia [6, 40]. It has been shown that a score of 3.62 is common for the general population [6], whereas a score of 3.59 was observed in the health care sector [41].

Oberrieder and colleagues [37] used the Bray Attitude Towards Obesity Scale (BATOS). For the BATOS, a score above 94 suggests negative prejudices towards obesity [42]. McArthur and Ross [36]; and Harvey et al. [34] used scales which included Likert-Scales to determine their level of agreement or disagreement regarding relevant statements (sample item: “Obese people are just as self-confident as other people” using a six-point Likert-Scale, ranging from 1 = “strongly disagree” to 6 = “strongly agree”).

In addition to prejudice and attitude, some studies also investigated information about perceived causes of adiposity. While Harvey et al. [34] directly asked participants what they believe is the reason for overweight and obesity using ten internal (e.g. “lack of willpower”) or external (e.g.” metabolic defects”) items, McArthur and Ross [36] indirectly examined dietitians’ beliefs by asking them about their own weight-related attitudes (e.g. “I attribute my excess weight to emotional problems” or “I am to blame for my excess weight”).

The study by Swift et al. [39] gathered data regarding participants’ estimation on how much adiposity is personally controllable or patients with obesity are responsible for it by using the BAOP scale (eight-items on a 6-point scale, scores range between 0 and 48, higher scores are an indicator of a strong agreement that obesity is not under an individual’s control).

### Stigmatizing Attitudes–explicit measures

Six out of seven studies showed significant weight-related prejudice by dietitians (students or professionals) towards obesity (Table 1). Studies by Berryman et al. [32], Puhl et al. [38], Hellbardt [35] and Swift et al. [39] used the FPS, reporting an average degree of fat phobia ranging between 3.35 and 3.8 (Table 2).

**Table 2:** Average FPS-Scores.

Study	Mean FPS-Score
Berryman et al. (2006) [32]	3.7
Hellbardt et al. (2014) [35]	3.35
Puhl et al. (2009) [38]	3.7
Swift et al. (2013) [39]	3.8
Sikorski et al. (2012) ^a^[6]	3.6
Sikorski et al. (2013) ^a^ [41]	3.56

Note: FPS = Fat Phobia Scale

^a^ for reference: average FPS of the study representing the German general population [6]; and health care professionals [41, 43]

Table 2 includes an attempt to compare our results to results of the general population and other health care professionals. Since a substantial number of studies used the Fat Phobia Scale as the main outcome measure, it is possible to compare FPS scores in different study populations. FPS scores ranged between 3.59 (HCPs) and 3.65 (general public) [6, 40], indicating slightly lower negative attitudes in dietitians and nutritionists in these particular German samples. However, conclusions from this review are mixed, since some studies showed higher FPS scores. In comparison to other HCPs, a study on attitudes of HCPs in general [43] found a mean FPS score of 3.16, suggesting that the studies that were summarized here (Table 2) show considerable weight stigma in dietitians and nutritionists. Berryman et al. [32] indicated that 16.0% of dietitians have strong negative attitude (FPS score: ≥ 4.4) and 13.0% have neutral, slightly positive attitude towards obesity (FPS score: ≤ 2.5). Similar results can be found in the study by Swift et al. [39], where 11.0% showed characteristics of fat phobia on a high scale, whereas only about 1.0% of all participants have a neutral to slightly positive attitude.

Table 3 summarizes specific characteristics and their prevalence.

**Table 3:** Systematic outline of studies summarizing characteristics attributed to individuals with obesity.

Attribution pair	Berryman et al. 2006^a^ [32]	Puhl et al. 2009^a^ [38]	McArthur & Ross 1997^a^ [36]	Hellbardt et al. 2014^b^ [35]
Lazy/ motivated	52.6%	41.0%		2.71 (n.s.)
bad / good				
No willpower/willpower	47.4%	41.0%		3.17 (***)
Unattractive/ attractive	47.4%	54.0%	18.5%	3.20 (***)
Poor self-control/discipline	60.5%	65.0%	42.6%	3.25 (**)
Insecure/secure	65.8%	80.0%		3.61 (***)
Poor self- esteem/self esteem	63.2%	75.0%	16.7%	3.63 (***)
Likes Food/dislikes food	89.5%	80.0%		3.67 (***)
Self-indulgent/ self-sacrificing	52.4%	47.0%		3.06 (**)
Overeats/undereats	81.6%	81.0%		3.51 (***)
Slow/fast	73.7%	68.0%		3.50 (***)
Inactive/active	71.1%	77.0%		3.47 (***)
Shapeless/shapely	68.4%	36.0%		3.56 (***)
no endurance/having endurance	63.2%	72.0%		3.50 (***)
Weak/ strong	36.8%	31.0%		3.02 (*)

Note: Vignette describing an overweight woman: 1 = positive attribute to 5 = negative attribute;

significance levels refer to the difference between the overweight vignette and a normal-weight vignette):

*p < .05,

**p < .01,

***p < .001

^a^agreement rate of characteristics about obesity is illustrated by percentages.

^b^mean scores for attribution of pairs of adjectives assigned to a

Items related to food (e.g. “likes food”) or physical activity (e.g. “inactive”/ “slow”) as well as “poor self-esteem” were found to have the greatest rate of agreement [32, 38] (Table 3).

Hellbardt et al. [35] found very negative scores for the following three pairs: “shapely/shapeless”, “insecure/secure” and “poor self-esteem/self-esteem”. Interestingly, they reported an overall FPS score of 3.35 using vignettes. The FPS score of a normal-weight vignette in this study was 2.61, being significantly more positive than the score of the obese vignette. Therefore, the authors found a negative evaluation of the obese vignette. Neutral to slightly positive views were reported by Harvey [34]. McArthur and Ross [36] reported that participants’ attitudes expressed towards individuals with overweight or obesity were rather ambivalent.

### Stigmatizing Attitudes–implicit measures

Edelstein and colleagues [33] investigated implicit prejudice towards obesity by using Implicit Association Testing, including the words “bad vs. good” as well as “motivated vs. lazy”. Implicit prejudice by dietitians towards obesity can be observed significantly (Table 1).

According to this study, 76.0% of dietitians under investigation had strong to moderate preferences for people without obesity or overweight compared to people with obesity. Interestingly, age seemed to have an effect on the results, as 87.0% participants aged 20-to-29-years and 80.0% of participants aged 30-to-39-years had strong to moderate preference for thin individuals, whereas out of the study group aged 40 or older, only 67.3% exhibited the same preference. Moreover, 85.2% of dietitians with an undergraduate degree and 75.0% of dietitians with a doctorate showed strong to moderate preference for thin individuals, compared to 67.2% of dietitians with a postgraduate degree.

### Causes and Attributions

Besides the aforementioned stigma and attitudes of dietitians, some studies also revealed presumed causes of obesity and indicated to what extent controllability and responsibility for obesity can be attributed. Table 4 summarizes the results of these studies.

**Table 4:** Summary of studies examining the dietitians’ believes about causes or controllability of obesity.

Study	Causes/Patient-blaming	Result
Harvey et al., 2002[34]	positive	Physical inactivity most important, followed by mood, eating too much of the wrong food, continuously dieting and interpersonal factors
Berryman et al., 2006[32]	(positive)	81.6% reported that “overeating” can be linked to obesity and overweight
Puhl et al., 2009[38]	(positive)	81.0% reported that “overeating” can be linked to obesity and overweight > according to the authors, the results suggest that participants tended to believe automatically that obesity is due to poorer diets and generally worse health (even when provided with information about individuals’ healthy lifestyle)
Swift et al., 2013[39]	positive	The belief that obesity is not under the individuals’ control was perceived stronger by students studying nursing compared to students studying Dietetics (the overall BAOP score including all students, was 13.4)
Hellbardt et al. 2014[35]	positive	Internal causes (e.g. overeating or lack of willpower and physical inactivity) were seen as more important than genetic factors or illness-related causes

Note: positive = patient is directly blamed as being responsible or having control over his/her weight; (positive) = patient is indirectly blamed as being responsible because the perceived causes of obesity are patient-centered.

As reported by Harvey et al. [34] physical inactivity and increased caloric intake due to unhealthy food are primarily named as underlying causes of obesity. Additionally, lack of willpower was thought to be rather important in causing obesity. On the other hand, reasons such as metabolic or genetic factors were undervalued. Interestingly, metabolic changes were assumed to be least relevant. The results obtained by McArthur and Ross [36] indicated that half of the dietitians base obesity on emotional problems and unrealistic goal setting by those affected. Although, dietitians did not show clear negative attitude in relation to people with obesity, in terms of their own weight, dietitians saw themselves as being responsible for it and, as the case may be, blamed themselves.

Hellbardt and colleagues [35] revealed that participants seemed to assess individuals as being responsible for their obesity (“lack of physical activity”, “overeating” and “lack of willpower”). Other factors, such as genetic reasons or illness-related factors (e.g. “metabolic disorder”) were seen as less relevant, which confirmed the opinion that obesity is a question of self-control only. Even if causes of obesity were not directly captured, Berryman et al. [32] and Puhl et al. [38] demonstrated similar reference to believed reasons within the Fat Phobia Scale, indicating that participants believed that the reason for obesity lies within the individuals’ area of control.

Swift et al. [39] applied the Beliefs about Obese People Scale) in order to determine to what extent students believe obesity as controllable. The analysis revealed that students studying dietetics are more likely to belief that obesity can be controlled by the person itself. The assumption that obesity is not under an individuals’ control was predicted by a smaller magnitude of fat phobia.

## Discussion

### Summary of Findings

The aim of this article was to review existing literature reporting the prevalence of weight-related stigma by dietitians and nutritionists (registered dietitians or students) towards people with overweight or obesity. Six out of eight studies under investigation reported prejudice by dietitians towards people with obesity, either on an explicit or an implicit level. Four studies that looked at attributions showed that overweight was seen as being manageable and that people with obesity were seen as being responsible for their excess weight and associated health conditions.

### Methodological Comparison

In terms of explicit prejudice the examined studies used questionnaires that differed in sensitivity, response modality, standardization, overall scores and quality criteria. McArthur and Ross [36] and Harvey et al. [34] developed a questionnaire that consisted of statements, which had to be classified as agreement or disagreement (on a scale), whereas others used standardized questionnaires such as Fat Phobia Scale in order to capture attitudes by using a list or pairs of adjectives. Furthermore, Hellbardt and colleagues [35] used two “weight-vignettes” in addition to the Fat Phobia Scale. However, the type of instrument used (standardized vs. self-constructed questionnaire) did not lead to different results.

Most studies in this review used explicit measurements only. Teachman and Brownell [44] also investigated whether health care professionals show weight stigma. They argued that the mere measurement of explicit prejudice was not sufficient due to their findings of greater variance in the explicit measurements compared to their implicit measurements In terms of explicit attitudes, people with overweight were seen as not “bad”, but “less motivated” than thin people, whereas for implicit measures negative attitudes were assigned to people with overweight in both cases equally. People might not be aware of their prejudice or they tried to be extensively fair and tolerant (social desirability) and therefore biased the overall results. The important effect of social desirability has been recently shown in a study by Azevedo et al. [45]. They found that perceived external (society-driven) pressure to act without prejudice was higher if participants knew about a hormonal disease as being the cause of obesity or overweight compared to a condition in which the reason for obesity was unknown. However, the internal motivation to act without prejudice towards obesity was not significantly different between the two groups.

Therefore, studies might be more reliable and valid, if they include both measurements. Despite the susceptibility of explicit measures, this review, however, demonstrated negative attitudes across most studies.

### Effects on Treatment

Previous literature on the prevalence of weight-related stigma in the health care sector has shown that it does not only affect physicians or therapists (e.g. [41, 46]), but also affects professionals that aim to treat or counsel patients with overweight or obesity. In summary, our findings suggest that even dietitians and nutritionists, who play a very important role in obesity management, may be prone to weight-related stigma. This can have reverse or negative effects on the treatment outcome and on the patient’s general physical and mental health (e.g. [4, 47]) leading to bad eating habits and reduced exercising [48].

Additionally, attitudes and beliefs could have affected practice choices made by dietitians [34]. They might for instance give advice on or focus on specific diets that include eating less only, ignoring other causes of obesity, such as genetic factors, failing to include systematic aetiology in their weight reduction strategy. In terms of motivation, weight-related stigma could also reduce encouragement and endurance of patients who try to lose weight. So clearly, if dietitians believe that their patients are just lazy, unmotivated or not able to set realistic goals, it will be difficult for them to plan strategies for their patients’ weight loss, provide enough support, have sufficient counseling skills, sympathy and caring attitude [36]. Seeing patients with obesity as competent and having positive attributes might do them good in terms of treatment seeking by having a beneficial effect on their self-image and hence in their weight reduction endeavors [26]. On the other hand, negative attitudes could be converted into negative treatment as suggested by another study [48] that shows that dietitians evolved positive feelings if their patients felt responsible for not being able to lose weight while dieting, compared to obese patients who blamed others for their failure, which in turn triggered adverse feelings. As a result, the researchers discovered 3 types of behavioral discrimination: instrumental avoidance (e.g. shorter meetings), professional avoidance (e.g. less effort) and interpersonal avoidance (e.g. negative tone or language). Patients who were perceived more positive for instance (because they blame themselves for their failed weight loss behavior were allocated more time with their dietitian compared to patients who were perceived more negatively.

### Determinants of weight stigma

A direct relationship between attitude and blame could not be found among all eight studies. On the one hand, three studies [35, 36, 38] tended to imply a link between ambivalent to negative attitudes and internally believed causes. Moreover, Swift et al. [39] showed negative attitudes in addition to the belief that overweight and obesity can be personally controlled. However, participants that were asked by Harvey et al. [34] believed that overweight and obesity was due to internal factors and can be controlled by the individual despite having neutral to positive explicit attitude. Therefore, there seemed to be no consistency between weight bias and allocation of blame towards the individual with obesity or overweight. Again, this question might need further investigation since it was hard to compare these studies that have not only been using different instruments and scales but also lacked measurements to reveal believed causes and controllability. The question still remains what the reasons behind weight stigma by dietitians and nutritionist are. Two other important determinants in this issue could be the amount of work experience gained in the field of treating obesity and overweight on the one hand, and the age of the professional on the other hand.

Interestingly, Puhl et al. [49] found that age as well as amount of experience might play an important role, since it was shown that older professionals with more experience in treating obesity expressed less weight bias- compared to, for instance, young professionals. According to Schwartz et al. [50] young adults were more affected by the societal pressure to be “in shape”, which increased during the last decades. Additionally, they argued that more negative prejudice toward individuals with obesity was a result of immaturity and lack of life experience. The same findings have been confirmed by three of the studies that have been discussed in this review [33, 35, 39]. However, some studies did reproduce neither the effect of age on bias [36] nor the effect of work experience or education on bias [33, 36, 37]. Another interesting factor, which might play a role in developing negative attitudes towards people with obesity or overweight, might be the professionals’ own weight. Two studies out of this review [37, 39] confirmed the assumption that a greater (self-reported) BMI is linked to less negative attitudes or lower fat phobia. Conversely, participants with a rather healthful weight tended to show more negative attitudes towards people with obesity and overweight. It could be argued that a deeper understanding of what it means to be overweight or obese due to personal experiences (with weight reduction attempts or even weight bias), might lower these negative attitudes. Moreover, personal BMI has been found to be one determinant of negative attitudes elsewhere [6].

Further research is needed to clarify this issue, including explicit as well as implicit measurements of weight-related stigma and a representative sample of dietitians and nutritionists with different sociodemographic backgrounds (e.g. age, work experience, BMI). In addition to that, it should be further investigated how perceived stigma might affect patients with obesity in general as well as their treatment outcomes. As mentioned above, weight bias in health care settings can result in impaired outcomes for patients with obesity and overweight, however, to our best knowledge this has not been investigated with regard to dietitians and nutritionists specifically.

### The origin of weight bias—a controversial issue

Most studies that were included in this review, argued that the first step should be to provide educational programs and interventions for those who want to professionalize in occupations aiming to help and support people with over-weight and obesity [36–39]. Moreover, weight stigmatization has been found to be directly linked to the belief, that obesity is due to behavioral factors rather than physiological or environmental causes in the general public as well [49]. Sikorski and colleagues [6] found evidence that believing in biological causes of obesity can be linked to lower negative prejudice towards these individuals. In other words, the knowledge of what causes overweight and obesity seems to be rather insufficient among the general public, but also among health care professionals [41]. Therefore, intervention programs that do not only focus on obesity management but additionally explain the aetiology behind overweight and obesity might improve attitudes by expanding the knowledge and expertise. Taking into account genetic or biological factors as causes of obesity might sensitize dietitians and nutritionists and enhance their understanding of their clients’ situation.

According to a review by Daníelsdóttir et al. [51], studies that tried to change prejudice and beliefs about reasons for obesity and whether it can be controlled by an individual, hence, reduce weight stigmatization, have been rather unsatisfying. It could be that stereotyping in relation to weight is firmly anchored -not only in adults, but also in children. Instead of reducing anti-fat bias (for instance by using intervention programs), medical explanations seem to amplify prejudice by provoking the need to avoid infection or disease. People might lack the understanding of the disease model of obesity, or might be negatively influenced by the overexposure to information provided by the media or other societal sources. Additionally, Tomiyama et al. [52] compared two sets of data from 2001 and 2013 and found reduced levels of implicit negative attitudes by health care professionals (including dietitians), but also revealed greater levels of explicit bias towards obesity. They debated that etiological knowledge about obesity was not conveyed into reduced weight stigmatization but rather increased explicit negative bias. This is in line with findings by Azevedo and colleagues [45], who based their results on fMRI-data in addition to explicit and implicit behavior measures. They found that stigma was more distinctive when participants knew about the aetiology of obesity (a hormonal disease) expressed by higher IAT scores and neuronal responses.

## Conclusions

So far, there seems to be a lack of sufficient evidence for reasonable approaches to reduce explicit as well as implicit negative attitudes towards obesity and overweight in society. To investigate if and why assumptions about causes of obesity and overweight might arise or change, could be the key to prevent weight-related stigma by dietitians and improve the health care condition for those that are stigmatized due to weight. It might be difficult to change society’s way of thinking about people with overweight or obesity but it could be a first step to start with the occupational group whose responsibility it is to treat them with understanding and respect in order to help them reduce any health risk that is related to their body weight. One way could be to include the issue of weight stigmatization (and its consequences for those affected) as part of the academic syllabus for students being educated in dietetics and nutrition as well as other related working areas. Intervention programs should not only focus on theory and scientific knowledge, but also call attention for discrimination and stereotyping. It might make them more sensible for this issue and therefore lower or efface their negative attitudes towards people with overweight or obesity. In addition to that it might help them prepare their patients in order to deal with weight bias in everyday life situations. Students as well as professionals should be made aware that mistreatment in terms of handling clients or patients as well as misunderstanding in regard of the aetiology of obesity can have negative effects on a physical or mental health level. Although there is mixed evidence whether intervention programs that aim to clarify the aetiology of obesity are helpful in reducing stigma, this component will need to be investigated more thoroughly in the future.

Weight stigmatization could negatively affect treatment outcomes or keep the patient from seeking medical advice. Patient-centered care does not only include functional skills and theoretical expertise- it is also about interaction and communication, motivation and patience, and probably most of all compassion and kindness.

## Supporting Information

S1 Fig PRISMA Flowchart. This flowchart summarizes why and how many studies have been excluded or included for further analysis.
(PDF)

S1 Table PRISMA 2009 Checklist. This checklist summarizes details about the methodological strategies that have been used to include or eliminate studies under review, for instance in order to overcome bias.
(PDF)
