# Supplementary figures and images for: Dietitians and Nutritionists: Stigma in the Context of Obesity. A Systematic Review
Source: PLoS One. 2015 Oct 14;10(10):e0140276. doi: 10.1371/journal.pone.0140276 (PMC4605484; doi:10.1371/journal.pone.0140276)

## PRISMA Flowchart

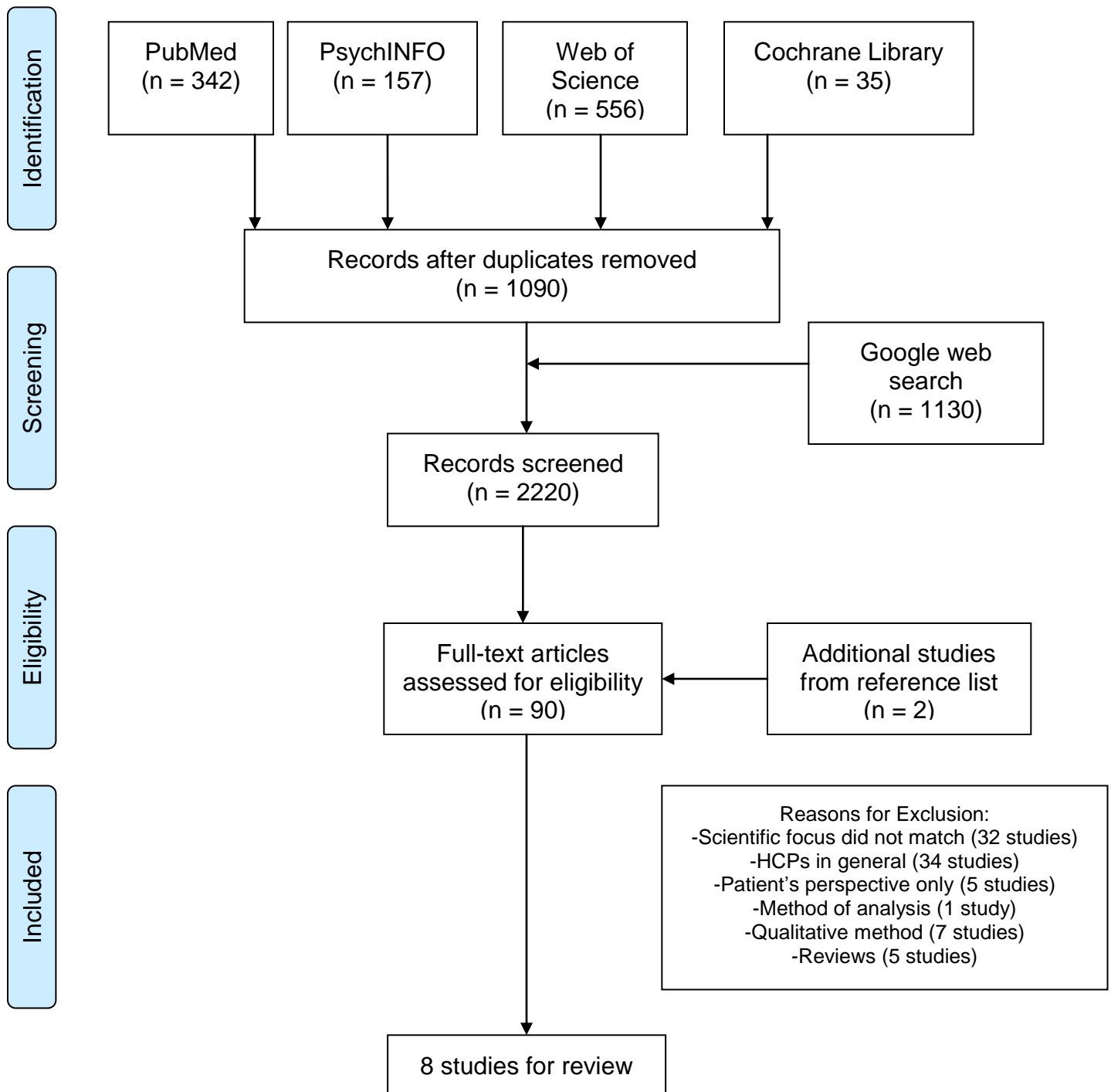

Supplement: S1 Fig — This flowchart summarizes why and how many studies have been excluded or included for further analysis. (PDF) [file pone.0140276.s001.pdf]
